# The Involvement of Semaphorins in the Pathogenesis of Skin Diseases

**DOI:** 10.3390/ijms242417235

**Published:** 2023-12-07

**Authors:** Sylwia Słuczanowska-Głąbowska, Olga Jankowska, Marzena Staniszewska, Andrzej Pawlik

**Affiliations:** Department of Physiology, Pomeranian Medical University, Powstańców Wlkp 72, 70-111 Szczecin, Poland; sglabowska@gmail.com (S.S.-G.); jankowskaolga.jo@gmail.com (O.J.); marzena.staniszewska@pum.edu.pl (M.S.)

**Keywords:** semaphorins, psoriasis, skin diseases, therapy

## Abstract

Semaphorins belong to a group of membrane and secretory proteins that act as ligands for several receptor families and are involved in modulating cell signaling pathways. They bind multimeric receptor complexes on the cell membrane to exert their effects and initiate unique intracellular signal transduction cascades. These proteins can influence several processes that are very important for cell function, such as cell division and differentiation. Semaphorins are involved in cell migration, apoptosis, cell adhesion, aggregation, and numerous immune processes due to their immunoregulatory effects. Semaphorins are expressed in keratinocytes, which is why they have become a target for studies on the pathogenesis of skin diseases. Most studies to date on the role of semaphorins in the pathogenesis of skin diseases have been carried out in cellular or animal models, and there are few clinical studies evaluating the role of semaphorins in the pathogenesis and therapy of skin diseases. In this narrative review, we summarized the current state of knowledge on the role of semaphorins in the pathogenesis of skin diseases and their potential importance as targets for therapy. We also tried to present the key findings and weaknesses of previous research in this field. The novelty of this article lies in the comprehensive presentation of the role of semaphorins in the pathogenesis of skin diseases, including the results of studies on cell cultures and animal models, elucidating the mechanisms and signaling pathways through which semaphorins affect the development of skin diseases, as well as on the presentation of the results of existing clinical trials evaluating the role of semaphorins in the pathogenesis of skin diseases, and as potential therapeutic targets.

## 1. Introduction

Semaphorins belong to a group of membrane and secretory proteins. More than 30 proteins have been identified and divided into eight classes in terms of structure and amino acid sequence. Classes 1–2 are semaphorins identified in invertebrates, classes 3–7 are in vertebrates and class 5 includes proteins found in the viral genome [1,2]. These molecules vary in structure but are characterized by a common, cysteine-rich Sema domain in the extracellular portion of the protein [1,2,3].

Semaphorins act as ligands for several receptor families and modulate cell signaling pathways. They bind multimeric receptor complexes on the cell membrane to exert their effects and initiate unique intracellular signal transduction cascades [1,2,3]. The main receptors for semaphorins are plexin-transmembrane proteins, in which four classes are distinguished. Most semaphorins bind directly to plexins, but some involve mediating proteins that are a type of coreceptor [4]. The signaling pathway induced by semaphorins leads to the remodeling of the actin cytoskeleton [4]. They bind plexin A, which has a highly conserved GAP domain (GTPase activating protein) and RBD domain (Rho GTPase binding domain) in their cytoplasmatic part. That leads to the activation of small G proteins belonging to the Ras and Rho families. Activated G protein stimulates GTPase activity by the acceleration of GTP dephosphorylation and GDP formation, which leads to signaling cascades. Proteins from the Ras and Rho families are known as regulators of migration, adhesion, differentiation, proliferation and survival of cells. Ras GTPases influence integrin function and control cell adhesion, whereas Rho proteins affect cell morphology and motility, playing a crucial role in actin cytoskeleton rearrangement. These changes influence cell morphology, cell–cell interactions, adhesion and migration of cells [5].

The most clinically relevant is Class 3 semaphorins [6]. Compared to other semaphorins, Sema3A has a wide range of expressions. Sema3A is a secretory protein that is secreted in many human cell types. It has been shown to be expressed in bone cells, connective tissue, kidneys, neurons, cartilage, cancer cells and human keratinocytes [7,8].

Sema3A consists of four structural domains. The N-terminal domain of Sema, the plexin–semaphorin–integrin (PSI) domain, the immunoglobulin-like (Ig) domain and the B (basic) domain at the C-end of the molecule [2]. The binding of class 3 semaphorin (Sema3A) to plexins occurs through another protein, coreceptor-neuropilin 1 (NRP1) [7,8]. NRP1 constitutes the receptor for Sema3A, while plexins transmit the signal further into the cell [2]. PlexinA and NRP1 form a stable complex. PlexinA alone does not bind Sema3A, but the NRP1/PlexinA complex has a higher affinity for Sema3A than NRP1 alone [9].

Signaling through Sema3A has been best understood in the nervous system as a molecule that directs neuron axon growth [2]. Sema3A, through the NRP1–plexin complex, shapes axons during neuronal development in embryogenesis. The interaction of Sema3A with neuropilins causes a halt in axon growth, known as growth cone collapse, which contributes to directing axons to their target areas [10,11]. Sema3A also possesses potent immunoregulatory properties [2]. Sema3A is considered to be a potent immunoregulator at all stages of the immune response, including both the early initiation and late phase of inflammatory processes [9]. Previous studies indicate that the immunomodulatory effect is mainly manifested by Sema3A’s effects on T lymphocytes, myeloid dendritic cells and macrophages. The most important effect of Sema3A is a suppressive effect on T lymphocytes [5]. Therefore, Sema3A has recently been recognized as a new paradigm in the pathogenesis of autoimmune diseases such as systemic lupus erythematosus (SLE), rheumatoid arthritis (RA) and multiple sclerosis (MS). Sema3A can reduce the symptoms of the aforementioned disease entities by suppressing the excessive activity of T and B lymphocytes and enhancing the regulatory properties of lymphocytes involved in the development of autoimmune diseases [11]. In autoimmune diseases such as SLE, RA and inflammatory bowel disease (IBD), reduced values of Sema3A were found in the blood in correlation with disease activity [3].

Sema3As are also Treg markers. In the co-culture of Sema3A with Treg cells, there was an increase in the number of Treg cells and an improvement in their function, suggesting that Sema3A may represent a promising new therapeutic strategy for treating autoimmune diseases [12,13,14]. That is shown in the research of Sema3A expression on Treg, serum levels of Sema3A, and tissue expression of Sema3A in bowel biopsies from patients with bowel diseases [12], on mouse models of rheumatoid arthritis [13] and the cells from SLE patients [14].

**Figure 1:**
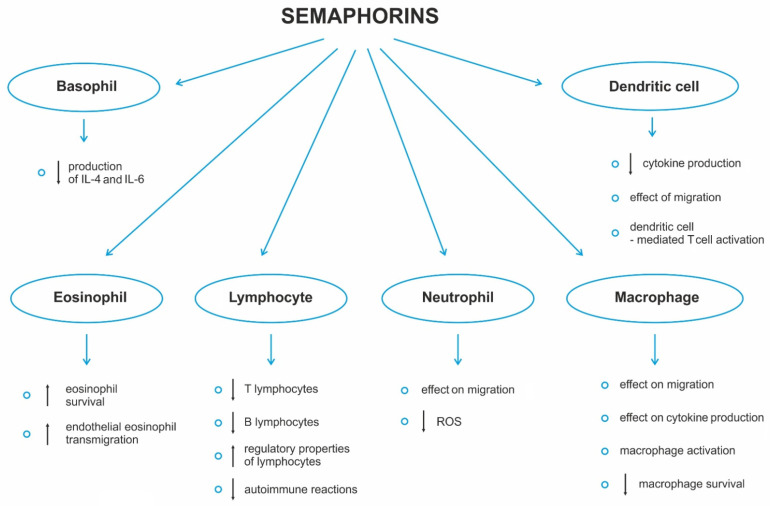
Multidirectional action of semaphorins.

Sema3A is believed to be closely associated with tumor progression and acts as a potent suppressor of tumor angiogenesis and VEGF function at various stages of cancer [13,14]. Sema3A competes with VEGF for binding to the NRP1/NRP2 coreceptors to inhibit the mitogenic effects of VEGF in endothelial cells, leading to apoptosis and inhibition of migration in breast cancer cell lines [15]. In hematopoietic and leukemic cells, Sema3A can also partially reverse the effects caused by VEGF [16]. The action of semaphorins is shown in Figure 1.

The regulation mechanisms of Sema3A expression in keratinocytes are still not well known. One of the research shows that Sema3A expression in normal human epidermal keratinocytes (NHEK) is induced via MAPK (mitogen-activated protein kinase) pathway, which includes MEK/ERK 1/2 kinases and activator protein (AP), mediated by calcium. The expression of Sema3A was increased in the presence of 0.1 or 1.4 mM calcium. On the other hand, calcium-mediated transient upregulation of Sema3A expression was significantly suppressed by inhibitors of this pathway (MAPK, ERK/MEK and AP-1 inhibitors). That confirms that expression of Sema3A is regulated by MAPK MEK/ERK1/2 kinases and AP-1 factor pathway under controlled conditions by calcium [17]. Another study confirms that Sema3A expression in NHEKs is elevated by high calcium concentration and reduced by low calcium conditions [18].

## 2. Involvement of Semaphorins in Skin Diseases

Previous studies have shown that semaphorins are involved in cell migration, apoptosis, cell adhesion, aggregation and numerous immune processes due to their immunoregulatory effects. Semaphorins are expressed in keratinocytes [4], which is why they have become a target for studies of the pathogenesis of skin diseases. They are found in the basal and suprabasal layers of the epidermis [4,19]. Most studies have been carried out on cellular or animal models, and there are few multicenter clinical studies evaluating the role of semaphorins in the pathogenesis and therapy of skin diseases. To date, the most widely studied semaphorin in skin diseases has been Sema3A. Studies indicate correlations between epidermal innervation and levels of Sema3A protein expression. The density of nerve fiber endings in the epidermis affects sensitization and the threshold for pruritus sensation in skin diseases [19]. As an axon guidance molecule, Sema3A inhibits the proliferation of sensory fibers [20,21]. Studies suggest that decreased expression of Sema3A accelerates epidermal nerve growth in patients with pruritic skin diseases [22].

Previous studies have shown that the density of epidermal nerve fibers is higher in the skin of patients with atopic dermatitis (AD), contact dermatitis and acute dry skin, compared to control subjects. Sema3A levels alone in the epidermis are lower in AD patients than in healthy subjects [19]. In acute dry skin, increased epidermal nerve fiber density has also been shown to correlate with decreased levels of Sema3A expression [20,21,22,23]. In addition, Sema3A affects nerve growth factor (NGF). Indeed, Sema3A has been shown to inhibit the induction of NGF-induced growth of afferent sensory fibers in the adult rat spinal cord. Therefore, it is believed that the density of nerve endings in the skin may be regulated by a balance between NGF and Sema3A [24].

One of the studies indicates the impact of glucose on Sema3A expression and epidermal nerve fiber density in the skin of diabetic patients. The common complication in diabetes is small fiber neuropathy (SFN), characterized by decreased intraepidermal nerve fiber density (IENFD). Study shows that Sema3A levels were remarkably increased in the suprabasal layer of the epidermis in either patients or rats with diabetes with SFN. The expression of Sema3A in diabetic keratinocytes was upregulated via mTOR-mediated p70 S6K and 4E-BP1 signaling pathways under high glucose conditions. That was concomitant with a reduced intraepidermal nerve fiber density (IENFD) seen in diabetic SFN. On the other hand, the upregulation was attenuated following rapamycin treatment. This research suggests that high glucose-upregulated Sema3A expression might contribute to the damage of intraepidermal nerve fibers (IENFs) and suggests that there might be a potential protective effect of the antagonists of Sema3A in diabetic SFN development [25].

Semaphorin 3A shows the interrelationships with antimicrobial peptides like Cathelicidins LL-37. This peptide upregulates the expression of Sema3A mRNA in cultured normal human epidermal keratinocytes (NHEK). LL-37 may bind to certain G10 coupled receptors, including P2X7R, activating the extracellular signal-regulated kinase signaling pathway and finally inducing Sema3A expression in human epidermal keratinocytes [26]. The induction of antimicrobial peptides, produced by epidermal keratinocytes, is impaired in lesional skin in patients with atopic dermatitis (AD), and decreased epidermal Sema3A production may be partially caused by a dysfunction in this pathway. Findings suggest that LL-37 may restore Sema3A production in keratinocytes of certain pathological conditions. The topical application of LL-37, which enhances Sema3A production, might be a useful therapeutic strategy for AD, especially accompanied by patients with intense itch symptoms [26].

Sema3A expression might be regulated by nuclear retinoid-related orphan receptor α (RORα) agonists. A study found that the cholesterol sulfate, which is an endogenous RORα agonist, dose-dependently upregulated Sema3A expression at both the mRNA and protein levels. Moreover, synthetic RORα/γ agonist SR1078 also upregulated the expression of Sema3A mRNA in cultured human keratinocytes. On the other hand, synthetic RORα/γ inverse agonist SR1001 inhibited Sema3A expression. Thus, ROR agonists may be used as a topical anti-pruritic treatment of skin diseases with epidermal hyperinnervation, such as AD, by inducing Sema3A expression [27].

Exogenous Sema3A, in complex with NRP1, affects not only sensory nerve fibers but also immune cells, endothelial cells and keratinocytes [28]. Intradermal administration of Sema3A improves skin condition at histological and macroscopic levels. Histological studies indicate that the application of exogenous Sema3A in lesional skin causes a decrease in the number of inflammatory infiltrates, including mast cells, eosinophils and CD4 T lymphocytes. IL-4 production and the density of cutaneous blood vessels and epidermal thickness also decrease [29,30,31].

In a study [29] on a mouse model of AD, the application of recombinant Sema3A by intradermal injection or ointment application, followed by a biopsy, showed significant improvement in the condition of lesions. In Sema3A-treated skin, a reduction in pruritus and a decrease in the number of nerve fibers in the epidermis were observed by immunohistochemistry. It is worth noting that the changes were observed only in the area treated with Sema3A. Sema3A acts locally and has a continuous effect for some time, even after discontinuation [29,30,31]. It is thought that replacement by exogenous Sema3A may have an anti-pruritic effect [20].

Several drugs are known to treat AD, but there is no targeted treatment, such as one that reduces the number of nerve fibers in the epidermis. Sema3A may effectively treat patients with AD who are resistant to the drugs used so far [29]. Treatment with anti-NGF agents and Sema3A substitution may normalize epidermal nerve fiber density. These findings may expand the knowledge of potential therapeutic strategies to alleviate pruritus associated with epidermal nerve density, including patients with AD and dry skin [20]. The therapeutic efficacy of exogenous Sema3A on AD symptoms in a mouse model has been greater than drugs such as betamethasone and tacrolimus [30]. Thus, Sema3A may be effective in AD patients refractory to existing treatments and in other skin diseases in which pruritus is present [20,29].

Several existing therapies can normalize abnormal levels of NGF and Sema3A in itchy skin associated with decreased epidermal nerve density. Oral administration of olopatadine hydrochloride, a receptor antagonist, significantly suppressed scratching, improved skin condition and inhibited neurite growth in the altered skin of mice with AD. Notably, olopatadine treatment increased Sema3A expression in the epidermis [28,32,33]. Olopatadine may partially improve the balance of NGF and Sema3A in the epidermis [20].

The next clinically important semaphorin is Sema4D. This protein is mentioned to have an impact on oral lichen planus (OLP) progression. Upregulations of Sema4D in OLP tissues and blood were positively correlated with disease severity and activity, whereas in healthy oral mucosa, Sema4D was nearly undetectable. Research showed that Sema4D has the capacity to drive CD8 T cells via chemokine modulation [34]. Sema4D binds plexin B1 via protein kinase B-N-kB cascade in human oral keratinocytes. That induces the production of chemokines C-X-C motif chemokine ligand 9 and C-X-C motif chemokine ligand 10 (CXCL9/CXCL10). In effect, that elicits CD8 T cell migration and recruitment, which maintains an autoimmune response in OLP [34].

Semaphorin 7A, unlike most semaphorins which inhibit neurite outgrowth, is known to stimulate axon growth. Sema7A stimulates melanocytes dendricity through the action of Plexin C1 and β1 integrin receptors, which are expressed on melanocytes. Plexin C1 activation has an inhibitory effect, but integrin β1 stimulates melanocyte dendricity. The regulation of melanocyte dendrite formation in the skin may be controlled through the inhibitory and stimulatory actions of Sema3A and Sema7A, respectively, as well as opposing signaling of integrins and Plexin C1 [35].

## 3. Involvement of Semaphorins in the Etiopathogenesis of Psoriasis

Psoriasis is a chronic inflammatory disease that significantly affects both the physical and psychological states of patients. Approximately 2% of the global population suffers from this disease, and it affects men and women equally. However, men are more likely to suffer from more severe forms. It can occur at any age but is most common between the ages of 20 and 40 and between 50 and 60 [36,37].

Symptoms are manifested primarily by inflammatory changes in the skin and joints [36]. The typical picture of the disease is well-demarcated, red, scaly plaques on the skin [4]. Light trauma (scratching, piercing, tattoos), sunburn, chemical irritants, infections—mainly *Streptococcus* infection, and drugs such as β-blockers, lithium, antimalarials, and NSAIDs—are considered triggers or exacerbators of the disease [38,39]. The occupational environment also affects the development or exacerbation of the disease if factors damaging the epidermal barrier are present [40]. Psoriatic lesions can affect any site on the skin; typical locations include the surfaces of the forearms and shins, the umbilical area, the anal area, and the retroauricular area and scalp. In addition to the skin, nails and joints are often involved in patients with psoriatic arthritis [41]. Psoriasis of the scalp develops in 75–90% of patients [42].

There are several types of psoriasis: plaque psoriasis (ordinary psoriasis), droplet psoriasis (papular psoriasis), pustular psoriasis and its subtype generalized pustular psoriasis, inverse psoriasis, and psoriatic erythroderma [36].

Psoriasis shows histopathological changes in almost all skin cell types. There is an abnormal proliferation of epidermal cells and a lack of apoptosis in keratinocytes [41]. Characteristics of the affected cells include epidermal acanthosis (the thickening of the squamous layer of the epidermis), hyperkeratosis (thickening of the keratinized layer) and parakeratosis (presence of cell nuclei in keratinocytes of the stratum corneum). T lymphocyte infiltration into the dermis and epidermis is present, as well as increased numbers of macrophages, mast cells and other granulocytes [42]. These cells accumulate in the epidermis to form what is known as Kogoja pustules or microabscesses, referred to as Munro’s microabscesses [36]. The pathogenesis of psoriasis is still not fully understood. It is a complex process involving genetic, environmental and immunological factors.

The involvement of semaphorin group proteins in the etiopathogenesis of psoriasis is currently an object of research. As in other skin diseases, previous work has analyzed Sema3A regarding its effect on keratinocyte proliferation, immune system inflammatory cells, and nerve fiber growth in the skin. Sema3A expression has been shown to decrease in the epidermis of psoriasis patients [43].

It is thought that Sema3A may play a significant role in psoriasis by affecting keratinocyte proliferation and VEGF, as it does in cancer cells. Keratinocytes in psoriasis show a characteristic of excessive growth, a process similar to that observed in cancer cells [43]. In one study, the effect of incubating a HaCaT cell culture with Sema3A and acitretin inhibited keratinocyte proliferation, migration, colony formation and induced apoptosis. The action of Sema3A and acitretin was also able to partially reverse the effect of VEGF on keratinocytes. These data support the hypothesis that Sema3A affects keratinocyte function and reverses the effects of VEGF [44]. Inhibition of keratinocyte migration by Sema3A has also been demonstrated [45]. Reduced expression of neurolipin 1 (NRP1) and Sema3A may contribute to the development of acanthosis, abnormal proliferation and differentiation of keratinocytes by facilitating cell migration [44].

Another aspect is the effect of Sema3A on immune cells. It is suspected that reduced expression of Sema3A in keratinocytes may modulate the immune response and facilitate the influx of inflammatory cells [45]. Sema3A and NRP1 are found in healthy skin and affect, among other things, the expression of Treg lymphocytes and the production of IL-10 and dendritic cells [44]. IL-10 is an anti-inflammatory cytokine produced by Treg lymphocytes, among others. To better characterize the function of Treg in psoriatic cells, it was shown that Treg cells are significantly altered compared to healthy skin cells, and IL-10 is downregulated [4]. Treg cells were found to be highly proliferative but qualitatively defective. The reduced expression of IL-10 in inflamed skin underscores the important regulatory role of this molecule in the pathogenesis of psoriatic skin lesions. In turn, the significant role of Treg is also supported by the fact that after phototherapy, along with an improvement in skin condition, the function of Treg cells improves, confirming the defective function of Sema3A/Treg in psoriasis-affected skin [4]. 

Studies at the molecular level demonstrate the effect of regulatory miR-142-3p in human keratinocyte cells of the HaCaT line on Sema3A gene expression. Repression of miR-142-3p leads to increased expression of the Sema3A gene, and this, in turn, alleviates psoriasis-like inflammation by inhibiting proliferation and promoting apoptosis of keratinocytes. It suggests that the miR-142-3p/Sema3A axis may be a novel therapeutic target for preventing abnormal keratinocyte proliferation and further confirms the correlation of Sema3A with inflammation [45,46]. A final important aspect of Sema3A function in the pathogenesis of psoriasis, as in other pruritic diseases, is its effect on nerve fiber growth and pruritus sensation. Pruritus affects 60–90% of patients with psoriasis and is accompanied by abnormal innervation of the skin [47]. The severity of pruritus correlates with the severity of the disease [44,46]. Several studies indicate a relationship between Sema3A expression and pruritic sensitization in psoriasis. It is suspected that reduced expression of Sema3A in keratinocytes of psoriasis patients may be involved in the development of pruritus [48,49]. The itch sensation in psoriasis is thought to be regulated by growth factors and nerve reduction. Factors that promote the growth of nerve endings are NGF and amphiregulin. The opposite effect is shown by Sema3A, which negatively affects the growth of neuromast fibers. In patients with psoriasis and AD, there is an increase in the expression of amphiregulin and NGF and a decrease in the expression of Sema3A in the epidermis compared to the control group [20,21,22,23,24].

**Figure 2:**
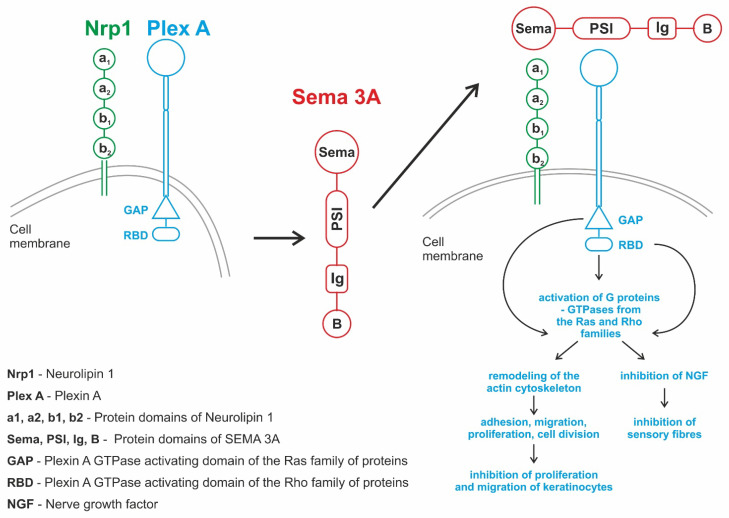
The involvement of semaphorin 3A in the pathogenesis of psoriasis.

In healthy skin, sensory fibers terminate at the junction of the dermis and epidermis. When Sema3A expression is downregulated, the fibers can ectopically press on the epidermis. The result is an increase in the innervation of the skin by penetrating the fibers into the epidermis. Both the intensity of pruritus and the clinical concerns of psoriasis show a negative correlation with Sema3A mRNA expression [50]. However, not all studies support the correlation of Sema3A with nerve fiber density in patients with psoriasis and pruritus. In one study [43], the results showed a trend but were not statistically significant. Nerve fiber density was higher in approximately 40% of psoriasis patients with pruritus compared to controls. Epidermal hyperinnervation was not found in all psoriasis patients with pruritus. Expression levels in the epidermis of Sema3A tended to decrease in psoriasis patients; however, the results were also not statistically significant [43]. Most work to date shows a significant effect of Sema3A on both keratinocyte proliferation and immune system inflammatory cells, as well as on the growth of nerve fibers in the skin. In many studies, both the intensity of pruritus and the clinical manifestations of psoriasis show a negative correlation with Sema3A mRNA expression. It is believed that Sema3A may play a beneficial role in the pathogenesis of psoriasis and other autoimmune diseases, and the administration of exogenous Sema3A may provide a new perspective for their treatment. The involvement of semaphorin 3A in the pathogenesis of psoriasis is shown in Figure 2.

The possibility of applying Sema3A to treat severe psoriasis when biological treatment fails to produce the desired results may prove to be a new therapeutic tool. Research to date has focused mainly on understanding the pro-inflammatory processes in the affected skin so that biological therapy can be used effectively. However, this is not always effective and partial remissions are not uncommon. Therefore, there has been a demand to study the regulatory rather than just pro-inflammatory mechanisms and to create a new therapeutic approach to act on the regulatory mechanisms [4]. 

**Figure 3:**
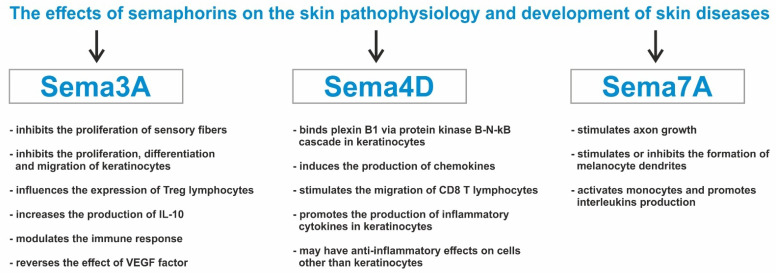
The effect of semaphorins on the skin pathophysiology and development of skin diseases.

Another semaphorin which might have an impact on psoriasis etiopathogenesis is Sema4D (also known as CD100). Researchers found out that the serum level of Sema4D is elevated in psoriasis patients and that the expression of Sema4D and its receptor PlexinB2 is increased in keratinocytes of psoriatic lesions. Sema4D-PlexinB2 complex promotes the production of inflammatory cytokines in keratinocytes by activating the NOD-like receptor protein (NRLP3) inflammasome. However, to confirm this thesis, they prepared a study research on a knockout Sema4D mice model. The skin inflammation of mice was significantly exacerbated in the early phase (2–8 days) compared to the wild type. In the chronic phase of the inflammation (10–16 days), there was no significant difference. The reason for this was the deficiency of Sema4D in dendritic cells. The study revealed a negative regulatory mechanism for dendritic cells in psoriasis through Sema4D-PlexinB2. Sema4D expressed on cells other than keratinocytes might play an anti-inflammatory function to overcome the pro-inflammatory role of Sema4D in the early phase of psoriatic dermatitis, and in the late phase, these two effects may reach a balance. That also confirms the fact that the expression level of PlexinB2 on dendritic cells from peripheral blood of psoriasis patients was lower than that on healthy individuals and that Sema4D inhibited the production of pro-inflammatory cytokines by dendritic cells. This specific targeting of Sema4D-PlexinB2 might be developed as a therapeutic strategy for the treatment of psoriasis [50]. The expression of PlexinB2 on keratinocytes is increased in the lesional skin of psoriasis patients, and the levels of Sema4D are elevated in sera of psoriasis patients and on keratinocytes of psoriatic skin. PlexinB2 binds Sema4D and creates a complex that promotes an inflammatory response. The complex activates the NLRP inflammasome and stimulates the NF-kB pathway in keratinocytes by activation of GTPase RhoA and Rac1. In effect, it promotes the production of various pro-inflammatory cytokines and chemokines and contributes to inflammatory response [51,52]. Next, the semaphorin, which may contribute to inflammation in psoriatic skin, is Semaphorin 7A (Sema7A). Sema7A reacts with β1 integrin expressed on monocytes, which induces monocyte activation and interleukin-8 production. The expression of Sema7A is regulated by various cytokines secreted by immune cells infiltrating into the upper dermis and epidermis. Study shows that TGF-β1 most strikingly increased Sema7A expression in keratinocytes [51]. The effects of semaphorins on skin pathophysiology and the development of skin diseases are shown in Table 1 and Figure 3.

## 4. Conclusions

Semaphorins belong to a group of membrane and secretory proteins that function as ligands for several receptor families and participate in modulating cell signaling pathways. Semaphorins can affect several processes that are very important for cell function, such as cell division and differentiation. Sema3A is involved in cell migration, apoptosis, cell adhesion and aggregation. Semaphorins are also involved in numerous immune processes due to their immunoregulatory effects. Sema3A is involved in various immune response processes, especially in initiating inflammatory processes. Previous studies indicate that semaphorins affect T lymphocytes, myeloid dendritic cells and macrophages. Therefore, semaphorins are believed to be an important element in the development of autoimmune diseases such as psoriasis. Most of the studies on the role of semaphorins in the pathogenesis of psoriasis have been conducted on animal models. So far, there is a lack of clinical studies evaluating the role of semaphorins in the pathogenesis of psoriasis. However, by evaluating the pathways of action of semaphorins, it seems that they may participate in the pathogenesis of psoriasis, as well as be a potential target for therapy. Current research is focused on understanding the exact role of semaphorins in the pathogenesis of psoriasis in initiating the development of the disease, modulating its course and affecting its activity and the development of systemic complications. It is very important to know the effects of semaphorins on signaling pathways related to the function of skin keratinocytes and on cells of the immune system involved in inflammation within the skin. It is also important to know the relationship of semaphorins to other pathways involving cytokines, chemokines and growth factors that modulate the function of skin keratinocytes. Studies are currently being conducted on the use of semaphorins for the treatment of severe psoriasis in patients who have not achieved improvement and remission of disease symptoms during the biologic treatment, but these studies have so far failed to provide sufficient evidence to support the therapeutic utility of semaphorins [33]. Preliminary results address the potential for topical use of semaphorin 3 [25,26]. However, taking into account the directions of action of semaphorins, it seems that they may be a promising therapeutic option, but this requires many clinical studies. The therapeutic use of semaphorins requires numerous studies, especially on the safety of such therapy and its possible side effects. Multicenter studies of the effects of semaphorins on the course of psoriasis, the activity and progression of the disease process and the occurrence of side effects are needed. Understanding the precise role of semaphorins in the development of psoriasis may contribute to a better understanding of this disease’s pathogenesis and bring new possibilities for its treatment. This requires several further studies, not only in animal or cell models but, above all, in multicentre clinical trials, to assess the role of semaphorins in the clinical course of psoriasis and the therapy of this disease.

## Figures and Tables

**Table 1:** The involvement of semaphorins in the development of skin diseases.

Type of Semaphorin	Skin Disease	Alterations Compared to Healthy Subjects	Major Effects on the Skin	References
**Sema3A**	Atopic dermatitis	decreased level of Sema3A expression in the epidermis	increased epidermal nerve fiber density	[19,29,30,31,48]
**Sema3A**	Acute dry skin	decreased level of Sema3A expression in the epidermis	increased epidermal nerve fiber density	[20,21,22,23,31,48]
**Sema3A**	Psoriasis	decreased level of Sema3A expression in the epidermis	- increased epidermal nerve fiber density - abnormal proliferation and differentiation of keratinocytes - modulation of immune response: e.g., higher influx of inflammatory cells; qualitatively defective Treg lymphocytes	[43,44,45,46,47,48,49]
**Sema4D**	Oral lichen planus	upregulations of Sema4D in tissues and blood	induction of chemokines production, leading to CD8 T cells migration and recruitment	[34]
**Sema4D**	Psoriasis	increased expression of Sema4D in tissues and blood	induction of inflammatory cytokines production in keratinocytes	[50]
**Sema7A**	Psoriasis	expression of Sema7A regulated by various cytokines	promotion of the inflammation (monocyte activation and IL-8 production)	[51]

## Data Availability

Not applicable.
